# Supplementary material for: Controllable field-free switching of perpendicular magnetization through bulk spin-orbit torque in symmetry-broken ferromagnetic films
Source: Nat Commun. 2021 Apr 30;12:2473. doi: 10.1038/s41467-021-22819-4 (PMC8087697; doi:10.1038/s41467-021-22819-4)
Supplement: Supplementary file 1 — Supplementary Information [file 41467_2021_22819_MOESM1_ESM.pdf]

Supplementary Information

Controllable field-free switching of perpendicular magnetization through bulk spin-orbit torque in symmetry-broken ferromagnetic films

Xuejie Xie[1,†], Xiaonan Zhao[1,†], Yanan Dong[1], Xianlin Qu[2], Kun Zheng[2], Xiaodong Han[2], Xiang Han[1], Yibo Fan[1], Lihui Bai[1], Yanxue Chen[1], Youyong Dai[1], Yufeng Tian[1,*] and Shishen Yan[1,*]

[1]School of Physics, State Key Laboratory of Crystal Materials, Shandong University, Jinan 250100, China
[2]Beijing Key Lab of Microstructure and Property of Solids, Institute of Microstructure and Properties of Advanced Material, Beijing University of Technology, Beijing 100124, China

[†]These authors contribute equally: Xuejie Xie, Xiaonan Zhao.
*Correspondence and requests for materials should be addressed to Y.T. (email: yftian@sdu.edu.cn) or to S.Y. (email: shishenyan@sdu.edu.cn).

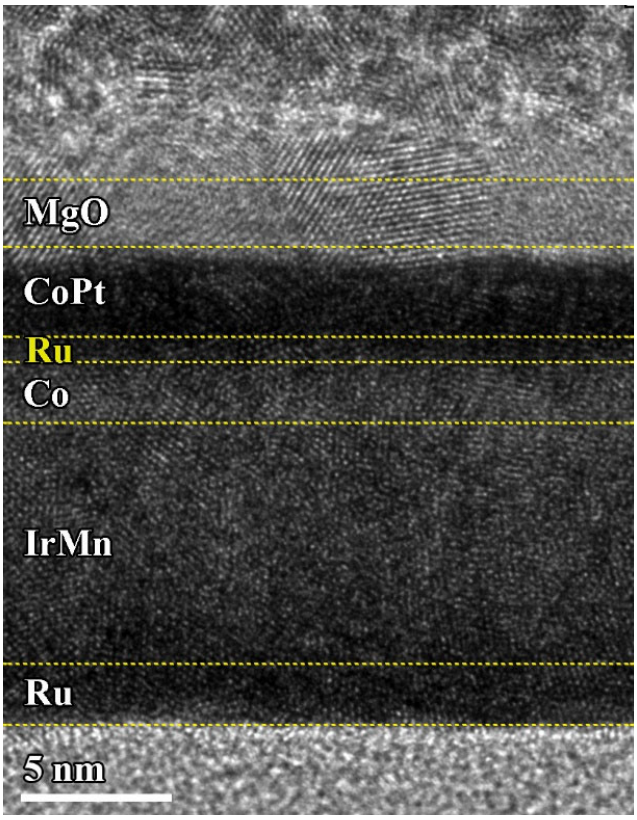

Supplementary Figure 1. HRTEM of the IrMn/Co/Ru/CoPt heterojunctions with the composition gradient CoPt alloy layer. It is clear that the single-layer CoPt alloy film was formed by the nominal multilayered structure of Pt(0.7)/Co(0.3)/Pt(0.5)/Co(0.5)/Pt(0.3)/Co(1).

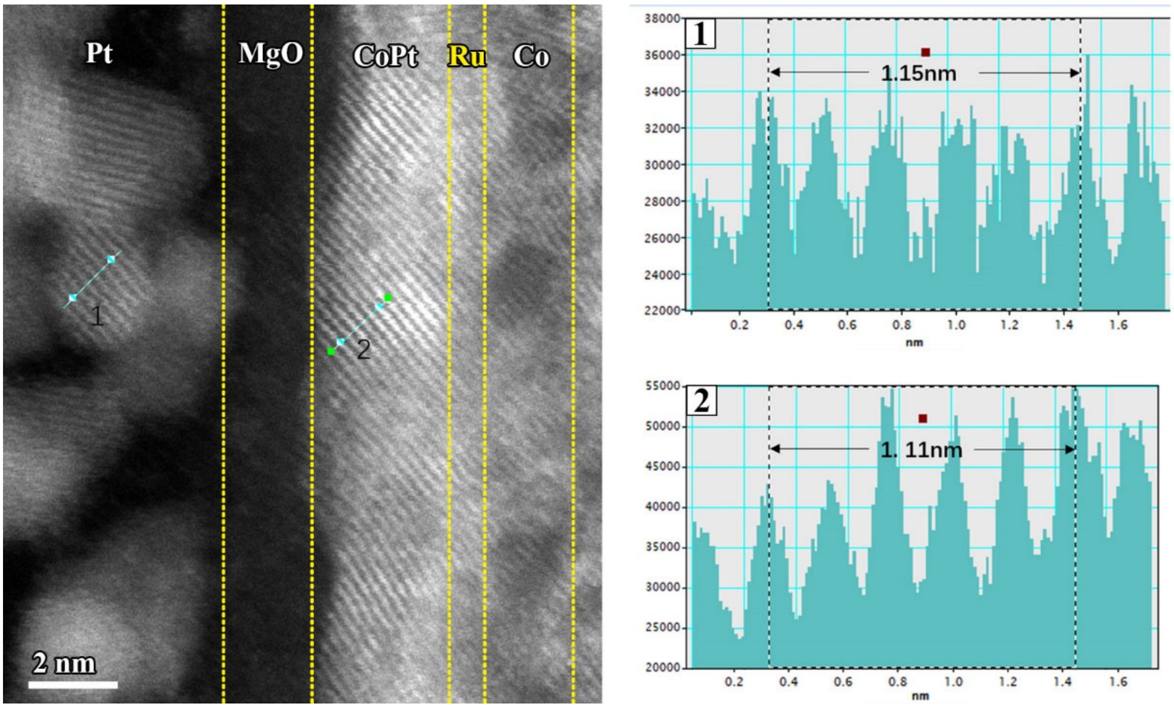

Supplementary Figure 2. HAADF images of the IrMn/Co/Ru/CoPt heterojunctions with the composition gradient CoPt alloy layer. Here, a pure Pt layer originates from the Pt coating for focused ion beam milling. Data analysis was performed with Digital Micrograph. It is easy to see that the lattice constant of CoPt (111) is slightly smaller than that of the pure Pt (111).

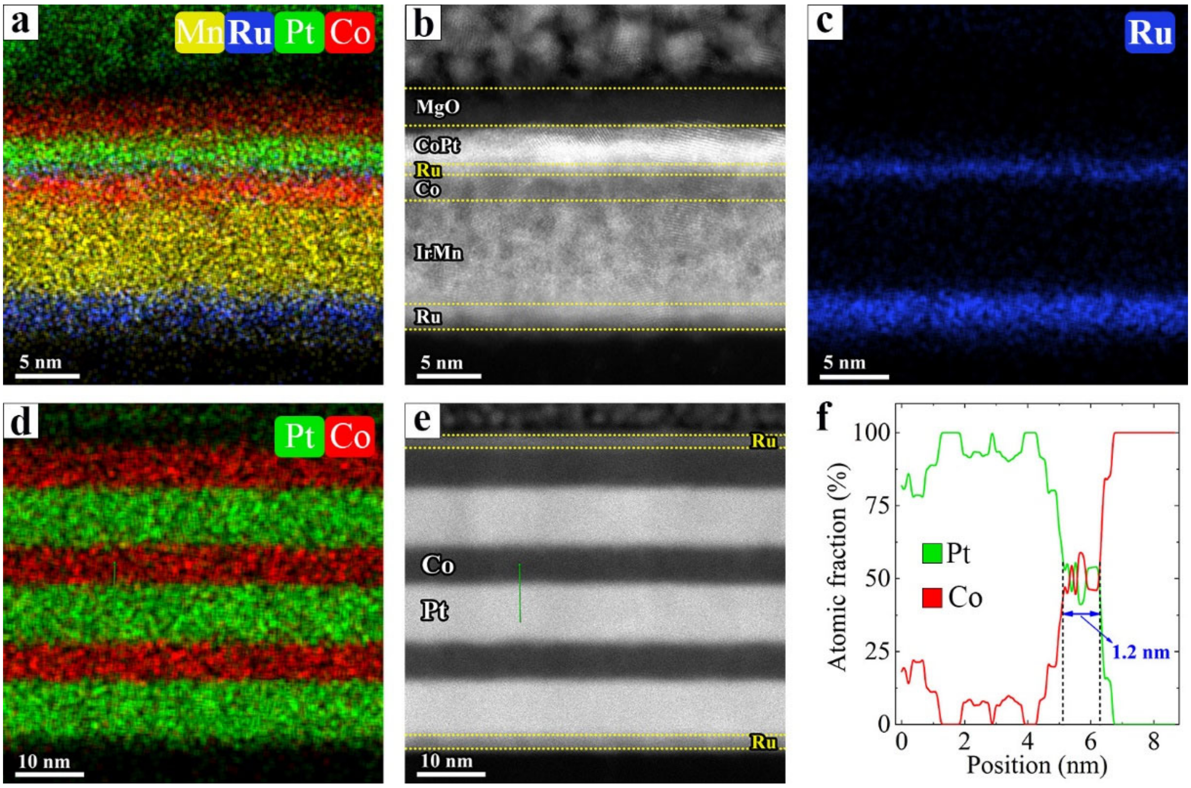

Supplementary Figure 3. Microstructure and composition characterization. a, The elemental mapping, **b**, the corresponding HAADF image, and **c**, the Ru mapping results of the studied IrMn/Co/Ru/CoPt heterojunctions. **d**, the elemental mapping, **e**, the corresponding HAADF image, and **f**, the line scanning of EDS results of the Ru(2)/[Pt(8)/Co(6)]$_3$/Ru(2) multilayer control sample. It clear shows that when very thin Pt and Co layers are alternatively sputtered for several times, CoPt alloy single-layer is formed due to the relatively strong atomic diffusion between Pt and Co. But the thin Ru layer of 0.8 nm is a separate and continuous sublayer in the IrMn/Co/Ru/CoPt heterojunctions, as shown in Supplementary Figure 3c. For the CoPt composition gradient layer, the top 1 nm Co layer is designed so that the composition gradient CoPt alloy layer has high Pt composition at the bottom and gradually evolves into the Co composition at the top, *i.e.*, the top Co layer of 1 nm is not fully alloyed with Pt. On the other hand, the top Co layer can be partially oxidized at the Co/MgO interface, which can further enhance the asymmetry of the CoPt composition gradient film. Therefore, a high Co composition is observed in the interface region of the Co/MgO interface, which shows some variation of contrast as compared with those of CoPt alloy and MgO in Supplementary Fig. 3b and Fig. 1e. However, the designed top 1nm Co layer does not influence the fundamental physics of SOT in the composition gradient CoPt alloy film.

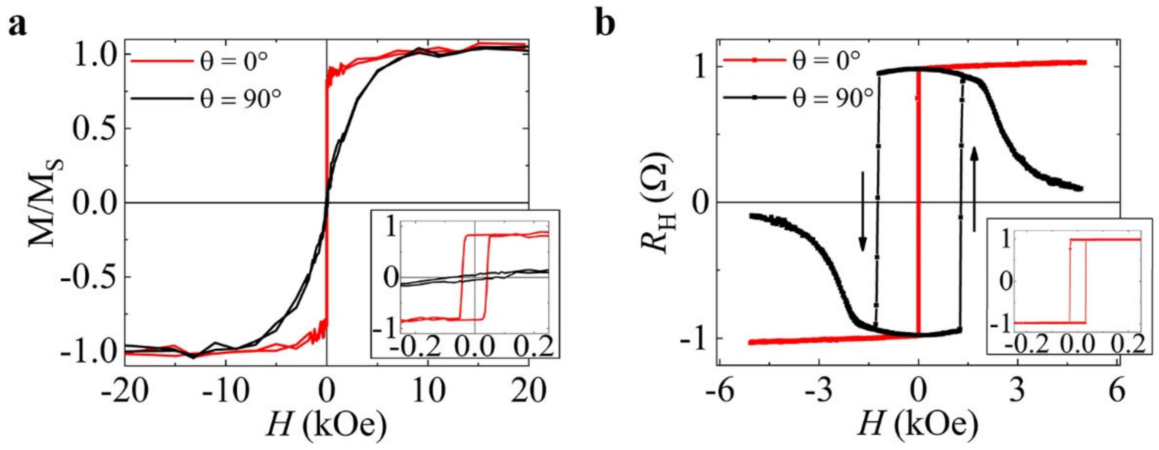

Supplementary Figure 4. Perpendicular magnetic anisotropy. a,**b**, The magnetic field dependence of the normalized magnetization M/M_{S} (**a**), and anomalous Hall resistance R_{H} (**b**) for both the out-of-plane ($\theta = 0°$) and in-plane ($\theta = 90°$) magnetic field. Here, θ represents the angle between the applied magnetic field and z direction. Insets show the enlarged view at low magnetic field.

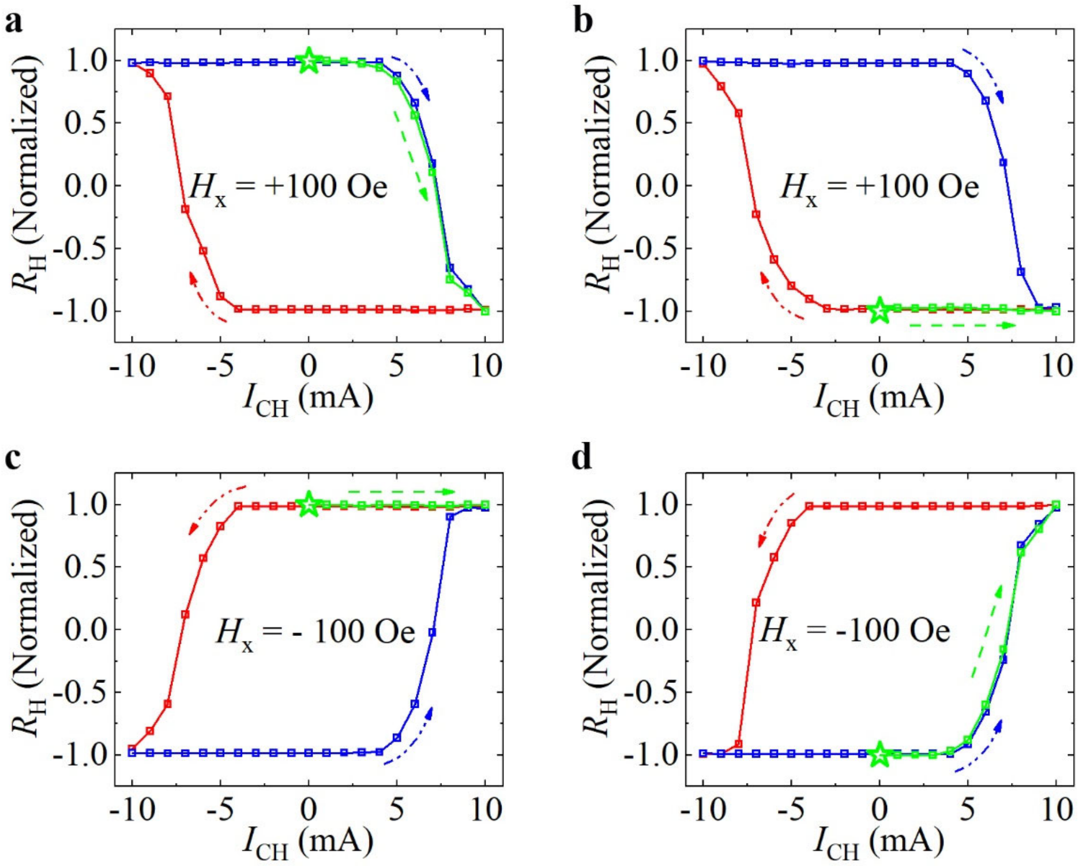

Supplementary Figure 5. Magnetization switching versus the initial magnetization states. a-d, Normalized $R_{\mathrm{H}} - I_{\mathrm{CH}}$ loops measured at different initial states under opposite magnetic fields of +100 Oe (**a**, **b**) and -100 Oe (**c**, **d**). The initial magnetization states (marked by green stars) were measured by using a small d.c. current of 0.1 mA before the current-induced SOT switching measurements were performed. The applied current sequence was divided into 3 parts: from 0 mA to 10 mA (green), from 10 mA to -10 mA (red) and from -10 mA to 10 mA (blue). It is observed that the switching polarity (clockwise or counterclockwise) of magnetization switching does not depend on the initial magnetization states.

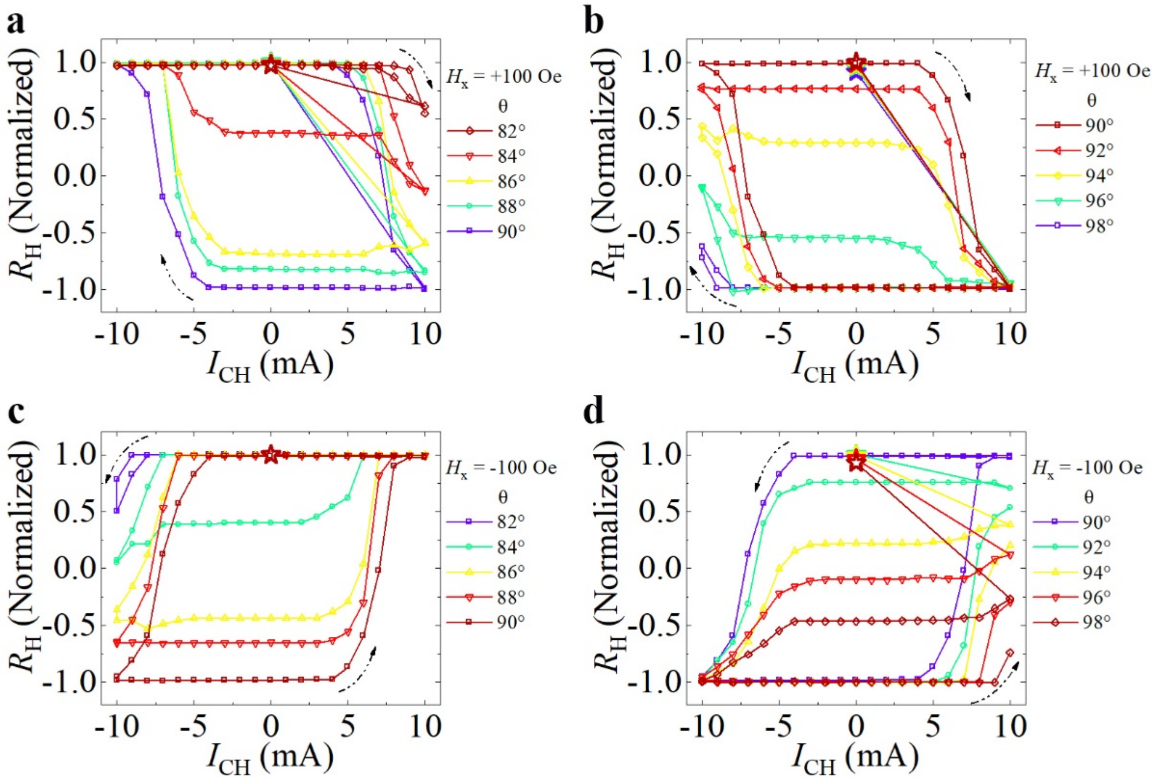

Supplementary Figure 6. The influence of geometric tilting on the observed SOT switching. a-d, Normalized $R_{\mathrm{H}} - I_{\mathrm{CH}}$ loops measured under $H_{\mathrm{x}} = +100$ Oe (**a**, **b**) and $H_{\mathrm{x}} = -100$ Oe (**c**, **d**) with different tilting angle θ, respectively. Here θ is the angle between the film normal direction and the external magnetic field. We can see that the switching polarity of magnetization switching is not affected by small geometric tilting.

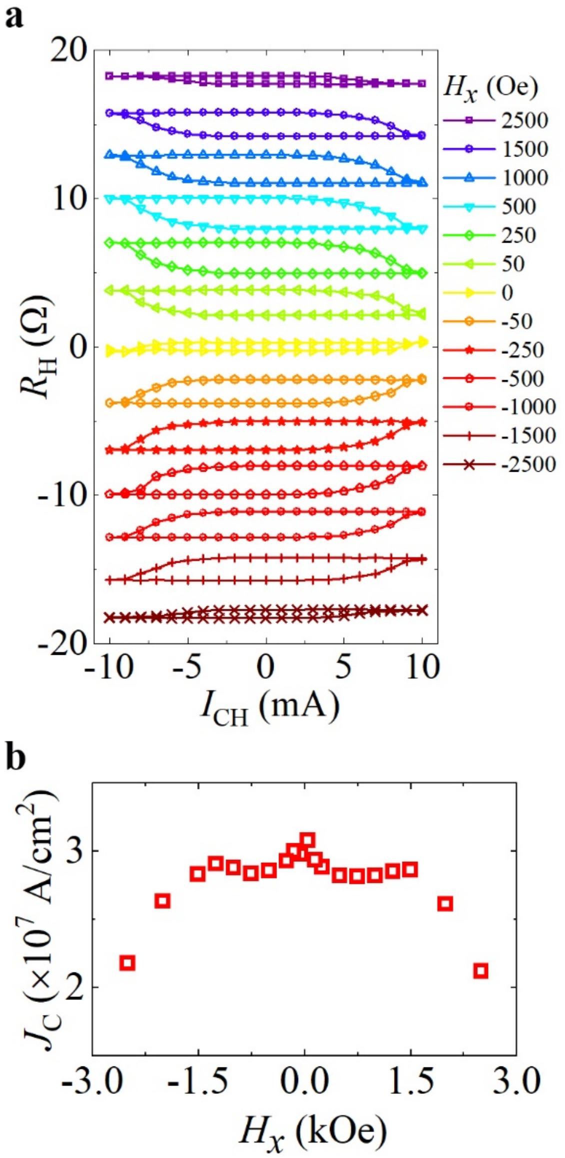

Supplementary Figure 7. Magnetization switching under various in-plane magnetic fields. a, $R_H - I_{CH}$ loops measured under various H_x. **b**, The H_x dependence of the critical switching current density J_C. Here, we scan electrical current ranging from -10 to +10 mA under a static H_x ranging from -2500 Oe to 2500 Oe.

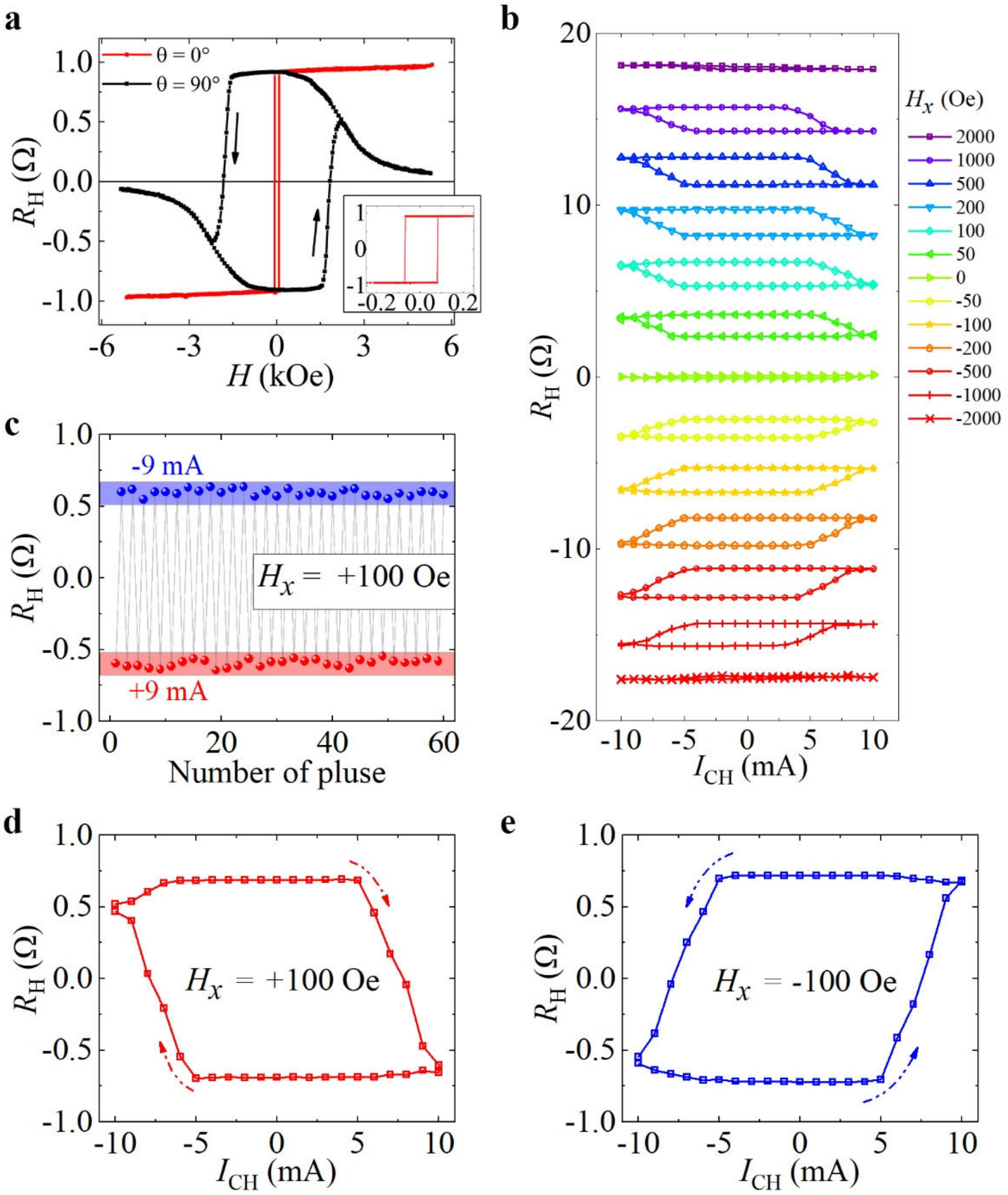

Supplementary Figure 8. Characterization of the control sample annealed at 300˚C for 1h. a, $R_{\mathrm{H}} - H$ loops for both the out-of-plane ($\theta = 0°$) and in-plane ($\theta = 90°$) directions, respectively. Inset shows the loop for the magnetic field range of ± 0.2 kOe. **b**, $R_{\mathrm{H}} - I_{\mathrm{CH}}$ loops under various H_{x}. **c**, Reversible magnetization switching induced by opposite pulsed current of ± 9 mA at $H_{\mathrm{x}} = +100$ Oe. **d**,**e**, respectively shows the $R_{\mathrm{H}} - I_{\mathrm{CH}}$ loops measured at $H_{\mathrm{x}} = +100$ Oe and $H_{\mathrm{x}} = -100$ Oe.

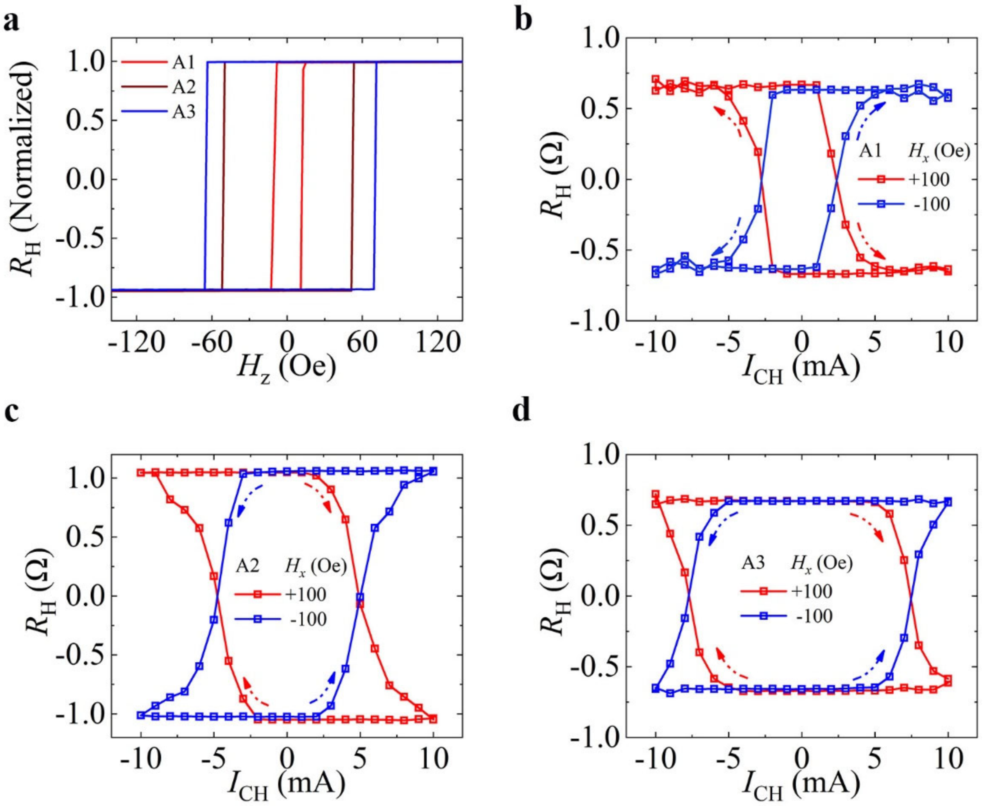

Supplementary Figure 9. Characterization of the control samples with positive Co composition gradient in the thickness direction. The core structure of the studied samples are given below.

A1: Pt(0.8)/Co(0.2)/Pt(0.5)/Co(0.5)/Pt(0.2)/Co(0.8);

A2: Pt(0.64)/Co(0.16)/Pt(0.48)/Co(0.32)/Pt(0.32)/Co(0.48)/Pt(0.16)/Co(0.64);

A3: Pt(0.49)/Co(0.21)/Pt(0.4)/Co(0.3)/Pt(0.3)/Co(0.4)/Pt(0.21)/Co(0.49).

The number in bracket is the layer thickness in nanometer. **a**, Normalized anomalous Hall resistance R_H versus the out-of-plane magnetic field for A1, A2 and A3, respectively. **b-d**, Current-induced magnetization switching in the presence of opposite in-plane magnetic fields H_x for A1 (**b**), A2 (**c**) and A3 (**d**), respectively. We can see that for the positive Co composition gradient and at the positive in-plane magnetic field H_x, the SOT switching polarity is always clockwise.

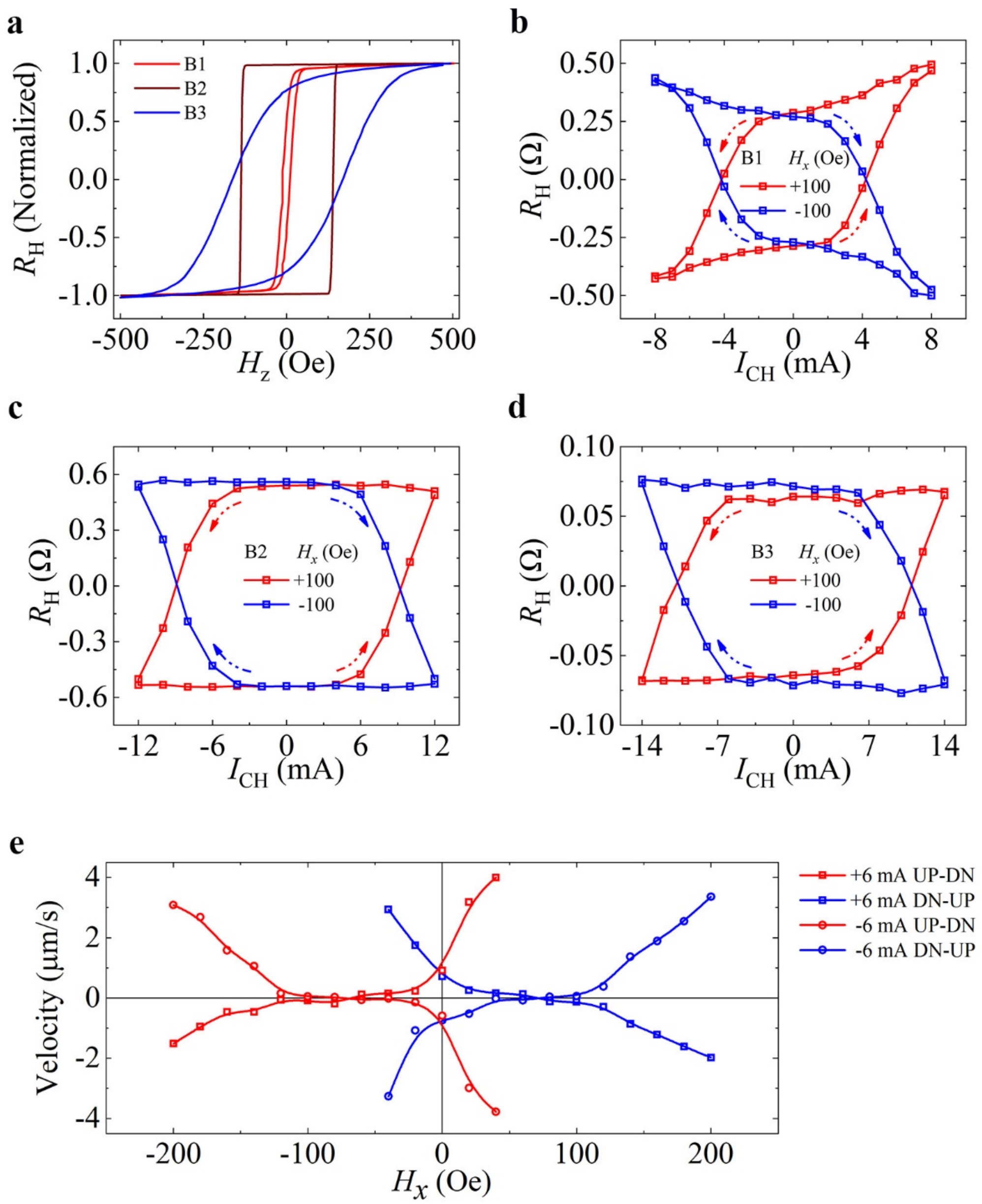

Supplementary Figure 10. Characterization of the control samples with negative Co composition gradient in the thickness direction. The core structure of the studied samples are given below, where the number in bracket is the layer thickness in nanometer.

B1: Pt(0.5)/Co(0.5)/Pt(0.65)/Co(0.35)/Pt(0.8)/Co(0.2);

B2: Pt(0.5)/Co(0.5)/Pt(0.6)/Co(0.4)/Pt(0.7)/Co(0.3);

B3: Pt(0.4)/Co(0.7)/Pt(0.5)/Co(0.5)/Pt(0.7)/Co(0.3).

a, Normalized anomalous Hall resistance R_{H} versus the out-of-plane magnetic field for B1, B2 and B3, respectively. **b-d**, Current-induced magnetization switching in the presence of opposite in-plane magnetic fields H_{x} for B1 (**b**), B2 (**c**) and B3 (**d**), respectively. **e**, Domain wall velocity versus in-plane magnetic field H_{x} for B2 sample. Red and blue symbols represent up-to-down (UP-DN) and down-to-up (DN-UP) DWs, respectively. Square and circular symbols correspond to positive and negative currents, respectively. We can see that from samples B1 to B3 the critical switching current monotonically increases with increasing the coercivity. However, for the negative Co composition gradient and at the positive in-plane magnetic field H_{x}, the SOT switching polarity is always counterclockwise. Comparing Supplementary Figs. 9 and 10, it is easy to find that the polarity of magnetization switching reverses when the composition gradient becomes opposite. Comparing Fig. 2f and Supplementary Fig. 10e, it is clear that the DMI effective magnetic field and the chirality of the DWs reverse when the composition gradient becomes opposite in the CoPt alloy films.

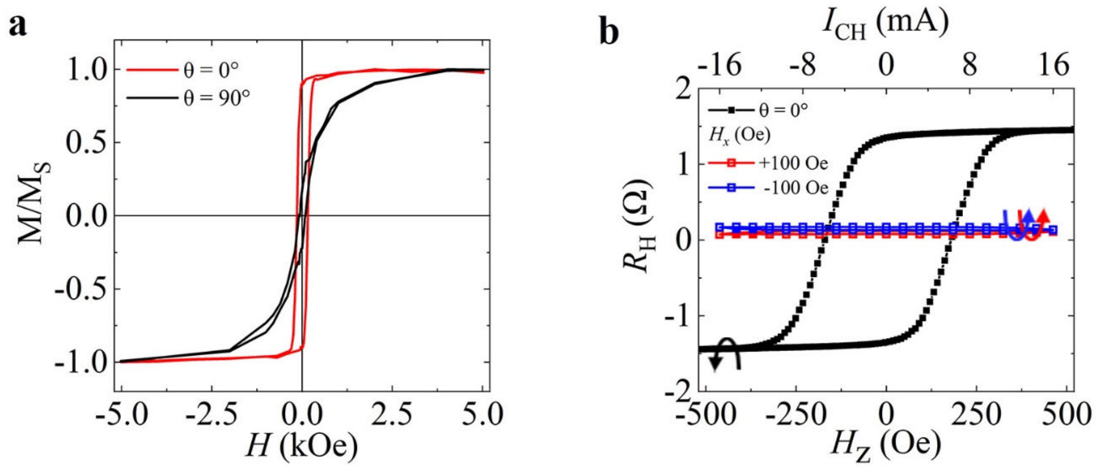

Supplementary Figure 11. Characterization of the Pt(2)/Co(0.5)/Pt(0.5)/Co(0.5)/Pt(0.5)/Co(0.5)/Pt(2) sample. **a**, The magnetic field dependence of the normalized magnetization M/M_S for both the out-of-plane ($\theta = 0°$) magnetic field and in-plane ($\theta = 90°$) magnetic field. **b**, Anomalous Hall resistance R_H measured under the out-of-plane ($\theta = 0°$) magnetic field (black) and the pulsed current at $H_x = +100$ Oe (red) and $H_x = -100$ Oe (blue), respectively. The sample has been set to the demagnetization states before the SOT-switching measurements. In the Pt(2)/Co(0.5)/Pt(0.5)/Co(0.5)/Pt(0.5)/Co(0.5)/Pt(2) sample with symmetric structure and no commposition gradient, it is found that although the perpendicular magnetization can be easily switched by the external magnetic field, it cannot be switched by the applied in-plane electrical current due to the symmetric structure. Here, the maximum applied current of 16 mA corresponds to a current density of 5.0×10^7 A cm^{-2}. Further increasing the applied electrical current may destroy the device.

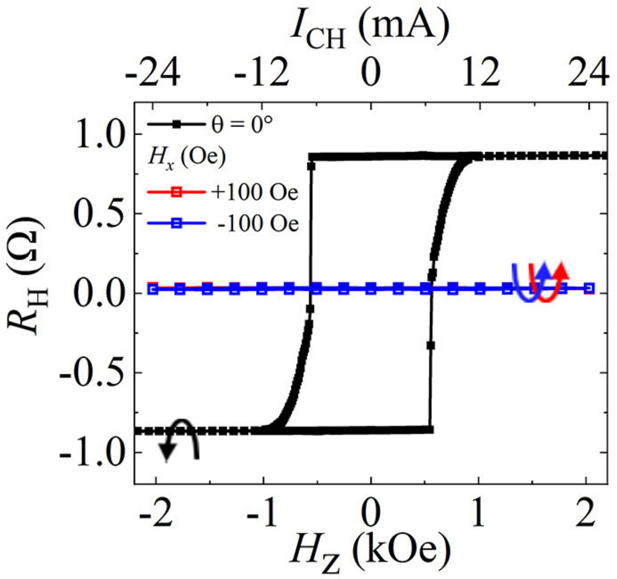

Supplementary Figure 12. Evaluation of the deposition indcued asymmetry. The Hall resistance (black curves) and the SOT-switching (red and blue curves) results of the Ru(2)/Pt(3.5)/[Pt(0.5)/Co(0.5)]$_8$/MgO sample. The sample has been set to the demagnetization states before the SOT-switching measurements. This sample has the deposition induced interfacial asymmetry but without composition gradient. Relatively thick [Pt(0.5)/Co(0.5)]$_8$ nominal structure is designed to reveal the possible bulk SOT effect. In Ru(2)/Pt(3.5)/[Pt(0.5)/Co(0.5)]$_8$/MgO sample, the spin Hall SOT of the 3.5 nm Pt layer and/or the Rashba effect of the CoPt magnetic layer due to the interfacial asymmetry can produce the longitudinal (ΔH_{L}, Slonczewski-like, along $\pm\hat{\mathbf{x}}$) and the transverse (ΔH_{T}, field-like, along $\pm\hat{\mathbf{y}}$) components of the effective fields on the single magnetic domains, *i.e.*, $\Delta H_{\mathrm{L}} \approx 4.0\ \mathrm{Oe}$ per $10^7\ \mathrm{A\ cm^{-2}}$and $\Delta H_{\mathrm{T}} \approx -0.54\ \mathrm{Oe}$ per $10^7\ \mathrm{A\ cm^{-2}}$, which were measured by first and second harmonic measurements. From the SOT-induced longitudinal effective field, we can extract the spin Hall angle $\theta_{\mathrm{SH}} \approx +0.066$, which is comparable to $\theta_{\mathrm{SH}} \approx +0.08$ of Pt layer. However, the magnetization switching can not be observed within the maximum electrical current of 24 mA, which corresponds to a current density of $4.5 \times 10^7\ \mathrm{A\ cm^{-2}}$. Further increasing the applied electrical current may destroy the device. This clearly indicates that only deposition induced interfacial asymmetry itself without the composition gradient can not lead to enough strong spin-orbit torque to switch the magnetization in our control samples with thick magnetic layer.

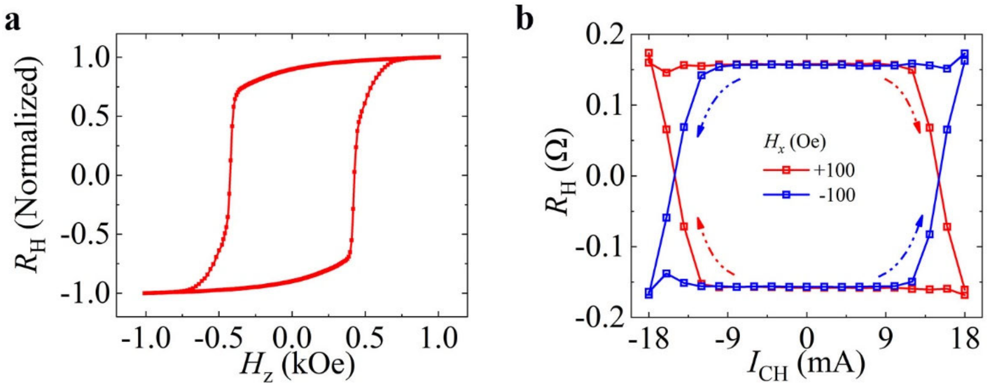

Supplementary Figure 13. Demonstration of bulk spin-orbit torque indcued magnetization switching in thick magnetic films. a, The normalized Hall resistance, and **b**, the magnetization switching of the $[Pt0.7/Co0.3/Pt0.5/Co0.5/Pt0.3/Co0.7/MgO0.3]_3$ sample measured under room temperature. Here, Pt0.7/Co0.3/Pt0.5/Co0.5/Pt0.3/Co0.7/MgO0.3 is used as a unit cell and a very thin MgO layer of 0.3 nm is chosen to enhance the perpendicular magnetic anisotropy while keeping the whole magnetic film to show magnetization switching as a single-layer film.

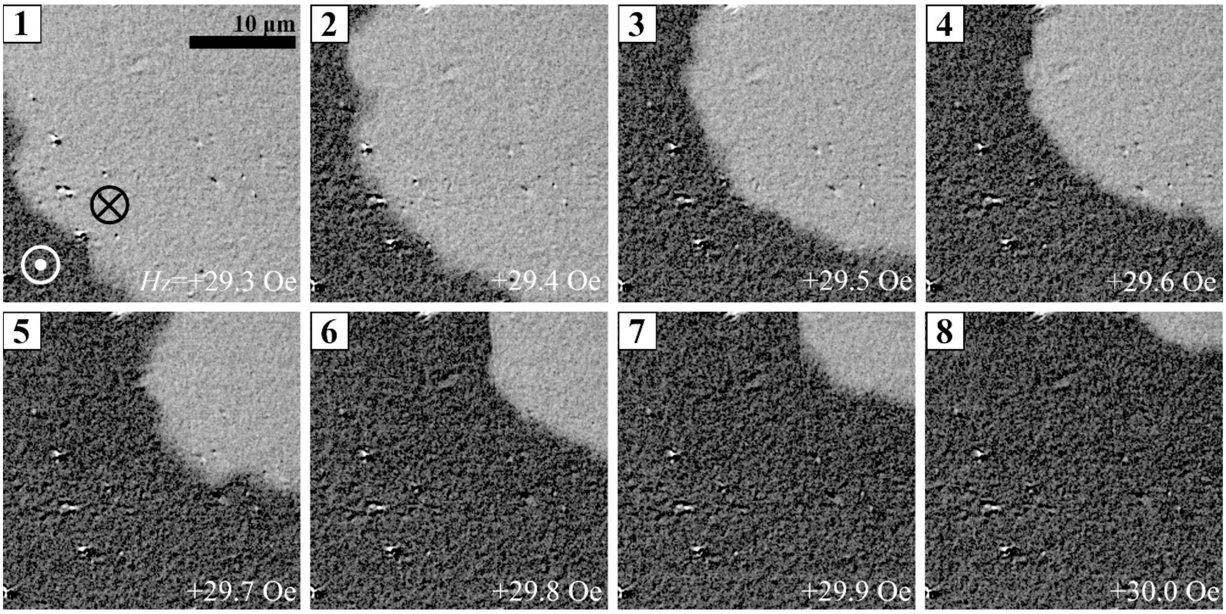

Supplementary Figure 14. The evolution of MOKE images of the CoPt composition gradient films with increasing the out-of-plane magnetic field $+H_z$. The bright and dark regions correspond to out-of-plane magnetization pointing down and up, respectively. The domain wall quickly moves from the up-magnetization domain to the down-magnetization domain as the magnetic field passed the coercivity. This clearly indicates that the magnetization reversal under the out-of-plane magnetic field $+H_z$ is caused by domain wall displacement.

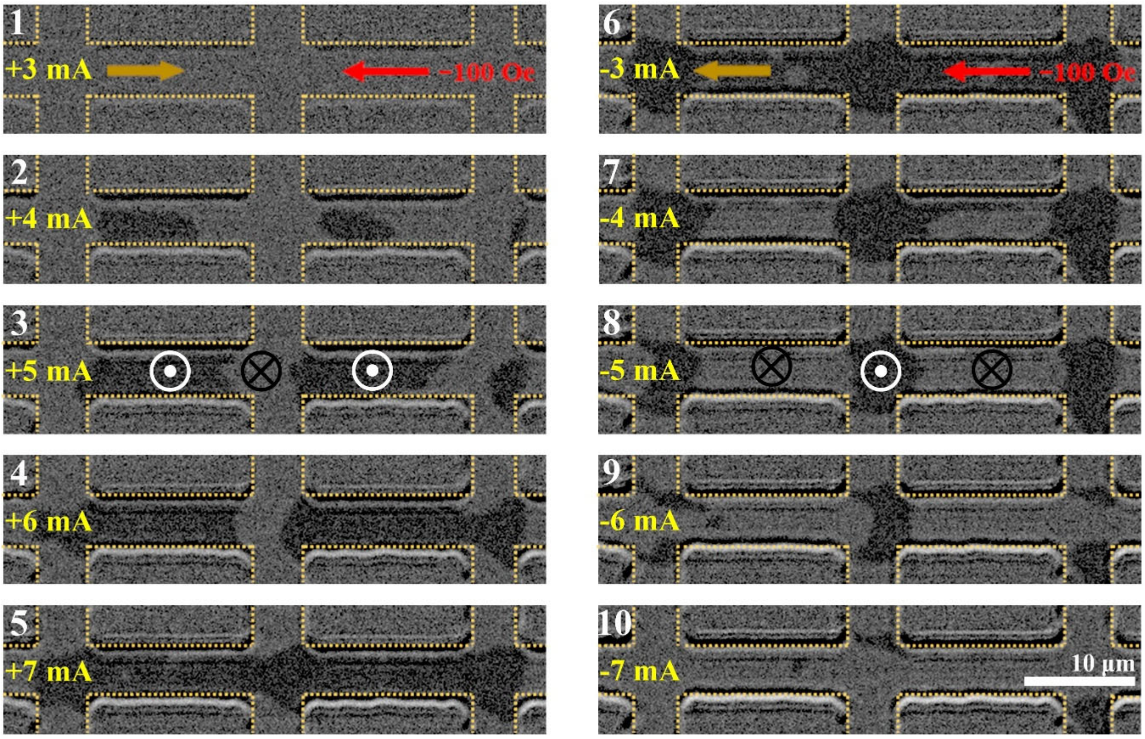

Supplementary Figure 15. MOKE images to show the current-induced magnetization switching process under a fixed in-plane magnetic field $H_x = -100$ Oe. The applied electrical current was scanned from -7 mA to +7 mA in the left column (only showing the images at +3, +4, +5, +6, +7 mA), and then scanned back from +7 mA to -7 mA in the right column (only showing the images at -3, -4, -5, -6, -7 mA). The yellow dashed lines indicate the Hall bar edges. The magnetization reverses from down to up as the applied electrical current was scanned from -7 mA to +7 mA, while it reverses from up to down as the applied electrical current was scanned from +7 mA to -7 mA. It is clear that the DWs nucleation and propagation induced by the external electrical current dominate the magnetization switching. From the magnetic domain evolution with time, we can obtain the domain wall velocity as a function of the applied magnetic field and/or the electrical current, as shown in Figs. 2e,f.

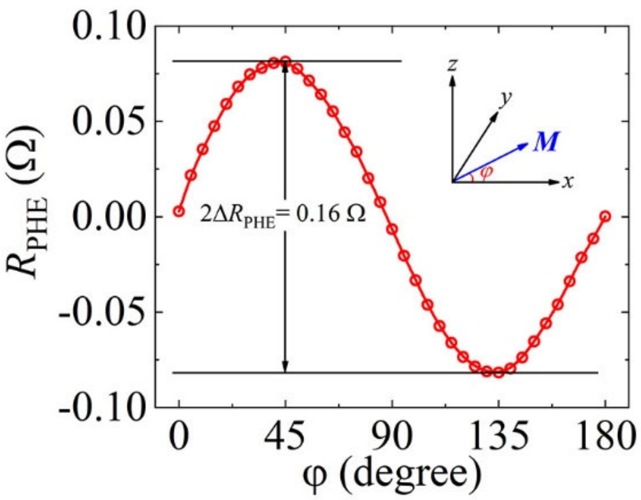

Supplementary Figure 16. The planar Hall effect of Ru/CoPt/MgO sample. The $R_{\mathrm{PHE}} - \varphi$ loop was measured under 5T in-plane magnetic field. φ is the angle between the magnetization **M** and x-axis in the x-y plane. $\Delta R_{\mathrm{PHE}} {\sim} 0.08\ \Omega$ can be obtained here, and the contribution from anomalous Hall effect is $\Delta R_{\mathrm{AHE}} {\sim} 0.98\ \Omega$, so the ratio ξ between ΔR_{PHE} and ΔR_{AHE} is about 0.082. Then the effective field-like and damping-like fields are corrected according to the following equation:

$$\Delta H_{\mathrm{L(T)}} = \frac{H_{\mathrm{L(T)}} \pm 2\xi H_{\mathrm{T(L)}}}{1-4\xi^2}.$$

Here, the $\pm$ sign refers to the ‘up’ and ‘down’ magnetized configurations, while ξ is the ratio between the planar (R_{PHE}) and anomalous (R_{AHE}) Hall resistances.

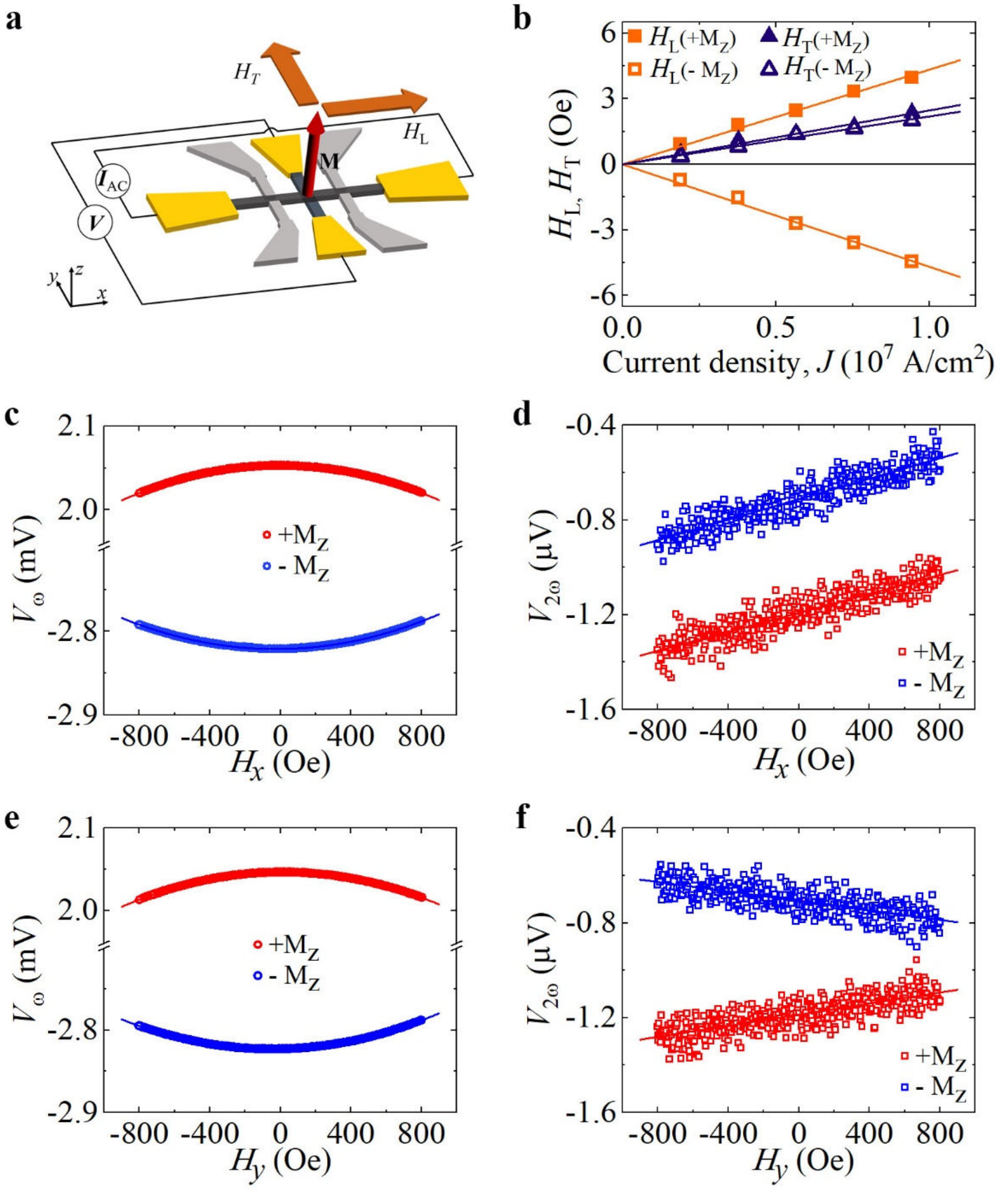

Supplementary Figure 17. Estimation of the effective field on the magnetic domains using harmonic Hall measurements. a, Schematic illustration of the effective fields (H_L and H_T) and measurement configuration. **b**, Calculated effective fields versus current densities. **c-f**, The external magnetic field dependence of first (**c**, **e**) and second (**d**, **f**) harmonic Hall voltage under an a.c. excitation with amplitude of 2.5 mA and frequency of 547 Hz. The magnetic fields H_x and H_y were swept along the $\pm$x (**c**, **d**) and $\pm$y (**e**, **f**) directions, respectively. The red and blue colors in (**c-f**) correspond to the $+M_z$ and $-M_z$ situation, respectively.

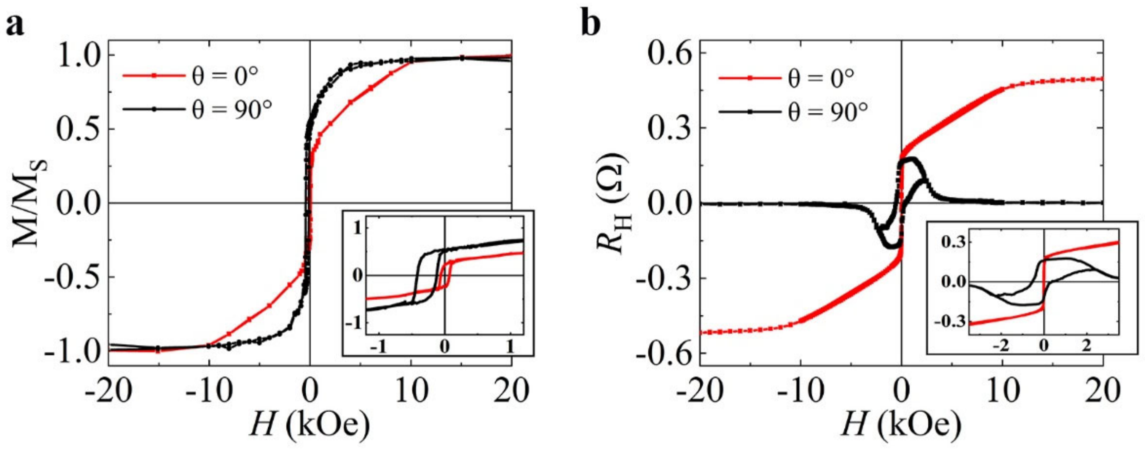

Supplementary Figure 18. Magnetization and Hall resistance of the IrMn/Co/Ru/CoPt heterojunctions. **a**,**b**, The normalized *M-H* curves (**a**) and anomalous Hall resistance (**b**) measured under both the out-of-plane ($\theta = 0°$) and in-plane ($\theta = 90°$) magnetic field. Insets show the enlarged view at low magnetic field. In the IrMn/Co/Ru/CoPt heterojunctions, the top CoPt alloy layer has perpendicular anisotropy, but the bottom Co layer have in-plane anisotropy. The bottom Co layer and top CoPt alloy layer are antiferromagnetically exchange coupled via a thin Ru spacer of 0.8 nm. In addition, the direct exchange coupling between the bottom Co layer and antiferromagnetic IrMn layer leads to a significant exchange bias field, which is consistent with the shift of in-plane magnetization hysteresis loops.

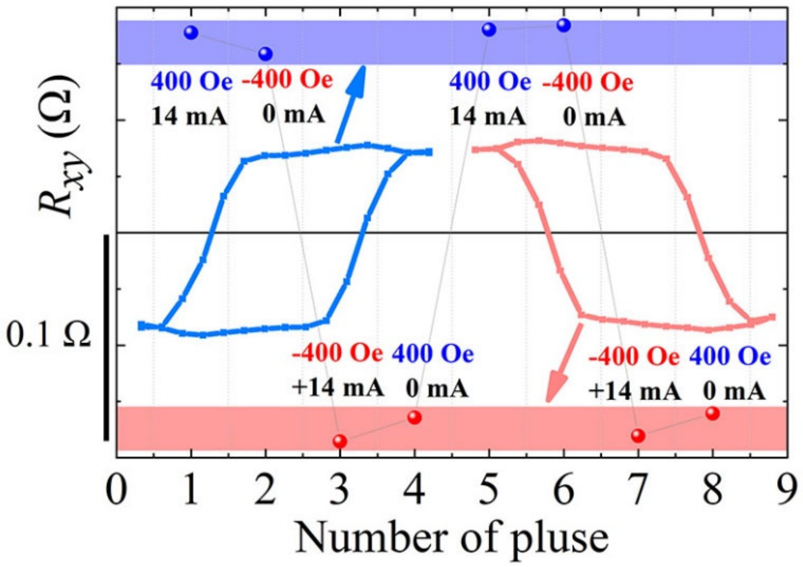

Supplementary Figure 19. Controllable switching polarity of field-free SOT switching in the IrMn/Co/Ru/CoPt heterojunctions, which was demonstrated by anomalous Hall resistance R_{xy} at zero magnetic field and corresponding reset parameters. The Hall resistance is measured at zero magnetic field after the reset magnetic field and current pulse were removed. The positive Hall resistance R_{xy} indicates the counterclockwise field-free switching after reset at $H_{\mathrm{x}} = 400\ \mathrm{Oe}$ and $I_{\mathrm{CH}} = +14\ \mathrm{mA}$. On the contrary, the negative Hall resistance R_{xy} indicates the clockwise field-free switching after reset at $H_{\mathrm{x}} = -400\ \mathrm{Oe}$ and $I_{\mathrm{CH}} = +14\ \mathrm{mA}$. However, the switching polarity of field-free SOT switching cannot be changed after reset at $H_{\mathrm{x}} = 400\ \mathrm{Oe}$ or $H_{\mathrm{x}} = -400\ \mathrm{Oe}$ without Joule heating.

Supplementary Table 1

The coercivity H_C, total metal layer thickness, critical switching current density J_{SW} at $H_x = +100$ Oe, the z component of the spin-orbit effective field on the magnetic Néel domain walls H_z at $H_x = +600$ Oe, the effective longitudinal (H_L) and transverse (H_T) field on the magnetic domains when the magnetization points to the $+M_z$ direction, and the calculated spin Hall angle θ_{SH}. --- means that the corresponding experimental data has not been obtained.

Sample	H_C (Oe)	Thickness (nm)	J_{SW} (10^7 Acm^{-2})	H_z (Oe/10^7Acm^{-2})	H_L $(+M_z)$ (Oe/10^7Acm^{-2})	H_T $(+M_z)$ (Oe/10^7Acm^{-2})	θ_{SH} $\times10^{-2}$
A0	31	5.3	2.80	9.5	5.0	3.0	1.0
A1	11	5.0	1.02	10.4	---	---	---
A2	52	5.2	1.87	19.5	17.92	8.11	1.97
A3	67	4.8	3.17	2.6	1.01	2.71	0.24
A4	425	11.0	2.74	5.0	2.89	-0.13	4.05
B1	12	5.0	1.67	-10.9	---	---	---
B2	138	5.0	3.61	-6.9	-7.23	0.81	-1.61
B3	166	5.1	4.25	-7.4	-2.09	-0.40	-0.45
C1	175	6.5	---	---	-4.27	-1.17	-3.08
C2	564	13.5	---	---	4.00	-0.54	6.61

The detailed nominal structure of the studied samples are given in the following:

A0: Pt(0.7)/Co(0.3)/Pt(0.5)/Co(0.5)/Pt(0.3)/Co(1),

A1: Pt(0.8)/Co(0.2)/Pt(0.5)/Co(0.5)/Pt(0.2)/Co(0.8),

A2: Pt(0.64)/Co(0.16)/Pt(0.48)/Co(0.32)/Pt(0.32)/Co(0.48)/Pt(0.16)/Co(0.64),

A3: Pt(0.49)/Co(0.21)/Pt(0.4)/Co(0.3)/Pt(0.3)/Co(0.4)/Pt(0.21)/Co(0.49),

A4: $[Pt0.7/Co0.3/Pt0.5/Co0.5/Pt0.3/Co0.7/MgO0.3]_3$,

B1: Pt(0.5)/Co(0.5)/Pt(0.65)/Co(0.35)/Pt(0.8)/Co(0.2),

B2: Pt(0.5)/Co(0.5)/Pt(0.6)/Co(0.4)/Pt(0.7)/Co(0.3),

B3: Pt(0.4)/Co(0.7)/Pt(0.5)/Co(0.5)/Pt(0.7)/Co(0.3),

C1: Pt(2)/Co(0.5)/Pt(0.5)/Co(0.5)/Pt(0.5)/Co(0.5)/Pt(2),

C2: $Pt(3.5)/[Pt(0.5)/Co(0.5)]_8$.

The A series samples (marked by red color) have a nominal positive Co composition gradient in the thickness direction, which means the Co concentration increases from the bottom to the top. The B series samples (marked by blue color) have a nominal negative Co composition gradient in the thickness direction. And the C series control samples (marked by green color) are designed to show no composition gradient

along the thickness direction. No reliable second harmonic Hall data is detected for samples A1 and B1 because of the small coercivity. The SOT switching polarity is always clockwise for A series samples and counterclockwise for B series samples at the positive in-plane magnetic field H_{x}, while no SOT switching is detected for C series samples, indicating that the composition gradient plays a critical role in the observed SOT switching. Moreover, it is clear that when the composition gradient is reversed the effective fields acting on both the magnetic domain walls (H_{z}) and magnetic domains (H_{L}) are reversed, resulting the opposite SOT switching polarity in the A and B series samples. However, a quantitative relationship between the composition gradient and the magnitude of the effect fields is hard to given at this stage, which require further systematic studies.
